# Association of 5-Hydroxytryptamine 3 Receptor Antagonists With the Prognosis of Liver Failure

**DOI:** 10.3389/fphar.2021.648736

**Published:** 2021-04-22

**Authors:** Yuting Chen, Jingkang Sun, Xiude Fan, Xiaoyun Wang, Lu Zeng, Xiaoge Zhang, Kun Zhang, Na Li, Qunying Han, Zhengwen Liu

**Affiliations:** ^1^ Department of Infectious Diseases, First Affiliated Hospital of Xi’an Jiaotong University, Xi’an, China; ^2^ Department of Postgraduate, Xi’an Medical University, Xi’an, China

**Keywords:** liver failure, 5-hydroxytryptamine 3 receptor antagonist, prognosis, short-term survival, treatment

## Abstract

Liver failure is a severe clinical syndrome with high mortality. 5-Hydroxytryptamine 3 receptor antagonists (5-HT3RAs) can reduce liver damage in animal models. We investigated whether 5-HT3RAs may improve the prognosis of liver failure. We analyzed the 28 and 90 days mortality of liver failure patients in relation to the use of 5-HT3RAs using data from a tertiary hospital in northwest China. According to the use of 5-HT3RAs, 419 patients with liver failure (46 acute, 93 sub-acute, 44 chronic, 236 acute on chronic) were divided into 5-HT3RA group (*n* = 105) and control group (*n* = 314). 5-HT3RAs were associated with decreased 28 days (HR 0.18, 95% CI 0.10-0.34, *p* < 0.001) and 90 days (HR 0.21, 95% CI 0.13-0.33, *p <* 0.001) mortality. After propensity score matching (PSM) (*n* = 67 in each group), 5-HT3RAs were still significantly associated with reduced 28 days (HR 0.10, 95%CI 0.04-0.26, *p* < 0.001) and 90 days (HR 0.16, 95%CI 0.08-0.31, *p* < 0.001) mortality. 5-HT3RA group patients had significantly higher 28 and 90 days survivals than controls both before and after PSM (all *p* < 0.001). This study shows that 5-HT3RAs are associated with increased survival of liver failure patients and thus may be used to treat liver failure if the findings are confirmed by additional studies.

## Introduction

Liver failure, a severe hepatic damage caused by various etiologies, is a devastating clinical syndrome of liver disease with many complications and high mortality. It manifests a group of clinical symptoms including jaundice, coagulation function disorders, hepatorenal syndrome, hepatic encephalopathy (HE), and ascites (Liver Failure and Artificial Liver Group, 2019). The treatment of liver failure is a challenging clinical problem due to the lack of effective approaches. Liver transplantation (LT) may improve the prognosis of liver failure (Lee et al., 2008; Artru et al., 2017). However, LT has many limitations, including the shortage of organs, surgical risks and the long-term use of immunosuppressants that may induce complications. Therefore, novel and alternative treatments are urgently needed for liver failure.

5-Hydroxytryptamine 3 receptors (5-HT3Rs), also known as the Cys-loop superfamily in eukaryotes, are cation-conducting pentameric ligand-gated ion channels found in the peripheral and central nervous system (Gaddum and Picarelli, 1997; Hargreaves et al., 1994). 5-HT3Rs are targeted by many therapeutic drugs such as the 5-HT3R antagonists (5-HT3RAs) that are widely used to prevent and treat nausea and vomiting caused by cancer chemotherapy, radiotherapy, anesthesia, and post-operation (Faerber et al., 2007). Anti-oxidative, anti-inflammatory and immunomodulatory properties have been observed for 5-HT3RAs (Fiebich et al., 2004a; Fiebich et al., 2004b; Stratz et al., 2012; Gong et al., 2019). At the same time, experiments in animals have indicated that 5-HT3RAs can alleviate liver damage induced by various causes (Fiebich et al., 2004a; Haub et al., 2011; Liu et al., 2011; Gholizadeh-Ghaleh Aziz et al., 2019; Amini et al., 2020).

In view of the anti-oxidative, anti-inflammatory and immunomodulatory properties (Fiebich et al., 2004a; Fiebich et al., 2004b; Stratz et al., 2012; Gong et al., 2019) and the protective effect on liver damage in animal models (Fiebich et al., 2004b; Haub et al., 2011; Liu et al., 2011; Gholizadeh-Ghaleh Aziz et al., 2019; Amini et al., 2020), we hypothesize that 5-HT3RAs may potentially influence the disease outcome in patients with liver failure. To this end, we performed a retrospective study to evaluate whether the use of 5-HT3RAs may affect the prognosis of patients with liver failure.

## Materials and Methods

### Study Population

This study included patients who were diagnosed with liver failure in the First Affiliated Hospital of Xi'an Jiaotong University from May 2013 to June 2019. The inclusion criteria in the patients were as follows: 1) age ≥18 years; and 2) clinical diseases met the diagnosis standards of “Guideline for diagnosis and treatment of liver failure (2018)” (Liver Failure and Artificial Liver Group, 2019). The exclusion criteria in the patients were as follows: 1) age <18 years; 2) pregnancy; 3) malignant tumor; 4) history of liver transplantation; 5) circulatory failure (Shock, congestive heart failure, etc.); and 6) incomplete data for analysis. The study protocol conforms to the ethical guidelines of the 1975 Declaration of Helsinki and the Ethic Committee of the First Affiliated Hospital of Xi'an Jiaotong University approved the study (XJTU1AF2020LSK-203) and waived the need of informed consent.

### Definitions

The definitions of liver failure are based on the “Guideline for diagnosis and treatment of liver failure (2018)” formulated by Liver Failure and ArtificiaI Liver Group, Chinese Society of Infectious Diseases, Chinese Medical Association; Severe Liver disease and Artificial Liver Group (Liver Failure and Artificial Liver Group, 2019). This Guideline referred the definitions from the Asian Pacific Association for the Study of the Liver (Sarin et al., 2014). Briefly, the criteria for the diagnoses of liver failures were as follows:

Acute liver failure (ALF): The occurrence of at least grade II HE within 2 weeks of the onset of symptoms in the absence of pre-existing liver disease with the following manifestations: 1) extreme fatigue, accompanied by severe gastrointestinal symptoms such as anorexia, abdominal distension, nausea, and vomiting; 2) progressively deepening jaundice [serum total bilirubin (TBIL) ≥10×upper limit of normal (ULN) or daily increase ≥17.1 μmol/l]; 3) hemorrhage tendency [prothrombin activity (PTA) ≤40%, or international normalized ratio (INR) ≥1.5 without other causes]; and 4) progressive liver shrinkage.

Sub-acute liver failure (SALF): The occurrence of the following manifestations from 2 to 26 weeks after the onset of the disease: 1) extreme fatigue and obvious gastrointestinal symptoms; 2) rapidly deepening jaundice (TBIL ≥10×ULN or daily increase ≥17.1 μmol/l); 3) with or without HE; 4) bleeding manifestations (PTA ≤40% or INR ≥1.5) without other causes.

Acute-on-chronic liver failure (ACLF): On the basis of a pre-existing chronic liver disease, the development of liver failure induced by various precipitating events or unidentifiable events and manifestation of acute jaundice deepening (TBIL ≥10 × ULN or daily increase ≥17.1 μmol/l), coagulation dysfunction (PTA ≤40% or INR ≥1.5), HE, ascites, electrolyte disorders, secondary infection, hepatorenal syndrome, hepatopulmonary syndrome, and the failure of extrahepatic organs.

Chronic liver failure (CLF): On the basis of liver cirrhosis, the development of progressive decline and decompensation of liver function with 1) increased serum TBIL (often <10×ULN); 2) decreased serum albumin (ALB); 3) decreased platelet count, and PTA ≤40% (or INR ≥1.5) without other causes; 4) manifestations of refractory ascites or portal hypertension; and 5) HE.

### Data Collection

Demographic, clinical, treatment, and laboratory data were collected from the electronic medical records. Demographic data included gender, age, ethnic (Han and non-Han), smoking, and alcohol drinking. Clinical data included etiology of the liver disease, classification of liver failure, hypertension, diabetes mellitus, nausea and vomiting symptoms, and complications (bleeding, ascites, infection, HE, hepatorenal syndrome, hepatopulmonary syndrome, electrolyte disturbance, and encephaledema). Treatment included artificial liver support treatment [plasma exchange (PE), plasma adsorption (PA), continuous renal replacement therapy (CRRT)], use of antivirals in hepatitis B virus (HBV) infected patients, anti-infective treatment, and the use of 5-HT3RAs. Laboratory data included liver and renal biochemistry, serum potassium, serum sodium, routine hematology, prothrombin time (PT), PTA, and INR. The model for end-stage liver disease (MELD) score was calculated in the patients based on laboratory data.

### Exposure to 5-HT3RAs and Outcomes

5-HT3RAs were used in patients with liver failure who needed symptomatic treatment for nausea and/or vomiting according to the judgment of clinicians. 5-HT3RAs used included ondansetron, granisetron, palonosetron, and tropisetron. Dosing regimes: ondansetron hydrochloride injection (4 ml: 8 mg), a single dose of 8 mg for intravenous injection (I.V.). Granisetron hydrochloride injection (3 ml: 3 mg), a single dose of 3 mg for I.V. Palonosetron hydrochloride injection (5 ml: 0.25 mg), a single dose of 0.25 mg for I.V. Tropisetron hydrochloride injection (5 ml: 5 mg), a single dose of 5 mg for I.V. The number of times of 5-HT3RA use was divided into <2 times or ≥2 times. The primary outcomes were 28 and 90 days mortality, defined as death or liver transplantation.

### Statistical Analysis

Categorical variables were described as frequencies (percentage) and were compared using the Pearson chi-squared test or Fisher’s exact test. Continuous variables were tested for normality. The normally distributed variables were described using mean ± standard and were compared using *t*-test. The non-normally distributed variables were expressed as the median (max, minimum) and were compared using Mann-Whitney U test. Patient 28 and 90 days survivals were estimated by Kaplan-Meier curves with log-rank tests for differences. In addition, collinearity between variables was detected by calculating the correlation coefficient and the *p* value between the variables. It was generally considered that the correlation coefficient >0.7, and *p* < 0.05 can be considered the existence of collinearity between the variables. In the two statistics of collinearity diagnosis of variables, namely tolerance and variance inflation factor (VIF), tolerance <0.2 or VIF >10 indicated that there was multicollinearity between variables. After calculation, the correlation coefficients between the variables in this study were all <0.7, tolerance >0.2 and VIF <10. Therefore, there was no collinearity between variables of this study. Cox proportional hazards regression model was used to assess the impact of all baseline covariates on the outcomes. In the regression model, hazard ratio (HR) and its 95% confidence intervals (CI) were calculated for each variable. Propensity score matching (PSM) was used to minimize the influence of data bias and confounding variables on the outcome. After calculating the propensity score, we used the 5-HT3RA group and the control group for patients with similar propensity scores using a 1:1 ratio matching, and the propensity score allowed the difference between the two groups to be 0.05. Inverse probability treatment weighting (IPTW) was also used. PSM and IPTW were performed used EmpowerStats 2.0 software, and the other statistical analyses used SPSS 25.0 statistical software. According to the test level of α = 0.05, *p* < 0.05 was considered as statistically significant.

## Results

### Characteristics of the Patients

During the study period, 509 patients were diagnosed with liver failure in the First Affiliated Hospital of Xi'an Jiaotong University. Of these patients, 67 patients were excluded from our study (5 aged <18 years, 1 pregnant, 54 with malignant tumors, 2 with a history of liver transplantation, 2 with circulatory failure, and 3 patients with incomplete data). There were 442 patients eligible for the study. After exclusion of 23 patients who were lost to follow-up, 419 patients were finally included in the study (Supplementary Figure S1). According to the use of 5-HT3RAs, the patients were divided into 5-HT3RA group (*n* = 105) and control group (*n* = 314, Table 1). PSM was performed to match all characteristics and 134 patients were selected (67 patients in each group, Table 1). The characteristics after IPTW were shown in Supplementary Table S1.

**TABLE 1:** Baseline characteristics in patients with and without the use of 5-HT3 receptor antagonists.

Variable	Before propensity score matching			After propensity score matching		
	5-HT3RA group (*n* = 105)	Control group (*n* = 314)	*p* value	5-HT3RA group (*n* = 67)	Control group (*n* = 67)	*p* value
Gender			0.093			0.213
Male, *n* (%)	65 (61.9%)	222 (70.7%)		45 (67.2%)	38 (56.7%)	
Female, *n* (%)	40 (38.1%)	92 (29.3%)		22 (32.8%)	29 (43.3%)	
Age (mean ± SD)	47.60 ± 14.55	46.98 ± 13.23	0.687	47.36 ± 14.85	50.43 ± 12.89	0.203
Ethnic			0.337			0.496
The han nationality	105 (100%)	309 (98.4%)		67 (100%)	65 (97.0%)	
Non-han nationality	0	5 (1.5%)		0	2 (3%)	
Etiology			0.104			0.589
HAV, *n* (%)	1 (1.0%)	0		0	0	
HBV, *n* (%)	61 (58.1%)	173 (55.1%)		38 (56.7%)	40 (59.7%)	
HCV, *n* (%)	0	4 (1.3%)		0	2 (3.0%)	
HEV, *n* (%)	0	3 (1.0%)		0	0	
Drug-induced, *n* (%)	13 (12.4%)	42 (13.4%)		10 (14.9%)	5 (7.5%)	
Alcoholic, *n* (%)	2 (1.9%)	23 (7.3%)		1 (1.5%)	2 (3.0%)	
Acute mushroom poisoning	0	1 (0.3%)		0	1 (1.5%)	
Severe infection, *n* (%)	6 (5.7%)	3 (1.0%)		6 (9.0%)	3 (4.5%)	
Autoimmune liver disease	2 (1.9%)	8 (2.5%)		1 (1.5%)	3 (4.5%)	
Cholestatic liver disease, *n* (%)	2 (1.9%)	7 (2.2%)		2 (3.0%)	2 (3.0%)	
Hepatolenticular degeneration	0	2 (0.6%)		0	0	
Budd-chiari syndrome, *n* (%)	0	1 (0.3%)		0	1 (1.5%)	
Unknown reason, *n* (%)	18 (17.1%)	47 (15%)		9 (13.4%)	8 (11.9%)	
Classification of liver failure			0.907			0.992
ALF, *n* (%)	10 (9.5%)	36 (11.5%)		9 (13.4%)	8 (11.9%)	
SALF, *n* (%)	22 (21.0%)	71 (22.6%)		12 (17.9%)	13 (19.4%)	
CLF, *n* (%)	11 (10.5%)	33 (10.5%)		6 (9.0%)	6 (9.0%)	
ACLF, *n* (%)	62 (59.0%)	174 (55.4%)		40 (59.7%)	40 (59.7%)	
Hypertension, *n* (%)	12 (11.4%)	20 (6.4%)	0.091	7 (10.4%)	7 (10.4%)	1.000
Diabetes mellitus, *n* (%)	3 (2.9%)	34 (10.8%)	0.013	3 (4.5%)	5 (7.5%)	0.718
Smoking status, *n* (%)	37 (35.2%)	136 (43.3%)	0.146	28 (41.8%)	25 (37.3%)	0.596
Drinking habit, *n* (%)	28 (26.7%)	107 (34.1%)	0.160	20 (29.9%)	17 (25.4%)	0.562
Nausea and vomiting symptoms	75 (71.4%)	55 (17.5%)	<0.001	38 (56.7%)	35 (52.2%)	0.603
Complication						
Bleeding, n (%)	42 (40.0%)	74 (23.6%)	0.001	25 (37.3%)	20 (29.9%)	0.360
Ascites, n (%)	75 (71.4%)	205 (65.3%)	0.247	45 (67.2%)	42 (62.7%)	0.587
Secondary infection, n (%)	91 (86.7%)	228 (72.6%)	0.003	58 (86.6%)	58 (86.6%)	1.000
HE, n (%)	34 (32.4%)	87 (27.7%)	0.360	22 (33.8%)	24 (35.8%)	0.716
Hepatorenal syndrome, n (%)	15 (14.3%)	21 (6.7%)	0.016	6 (9.0%)	9 (13.4%)	0.411
Hepatopulmonary syndrome n (%)	1 (1.0%)	1 (0.3%)	0.439	0	0	-
Electrolyte disturbance, n (%)	42 (40.0%)	118 (37.6%)	0.659	27 (40.3%)	22 (32.8%)	0.370
Encephaledema,n (%)	4 (3.8%)	13 (4.1%)	1.000	3 (4.5%)	3 (4.5%)	1.000
Artificial liver support treatment						
PE, n (%)	53 (50.5%)	104 (33.1%)	0.001	29 (43.3%)	26 (38.8%)	0.598
PA, n (%)	8 (7.6%)	32 (10.2%)	0.438	4 (6.0%)	3 (4.5%)	1.000
CRRT, n (%)	17 (16.2%)	27 (8.6%)	0.028	9 (13.4%)	10 (14.9%)	0.804
Antiviral therapy, n (%)	61 (58.1%)	167 (53.2%)	0.382	41 (61.2%)	36 (53.7%)	0.382
Antiviral drugs used			0.574			0.508
ETV, n (%)	51 (48.6%)	144 (45.9%)		29 (43.8%)	33 (49.3%)	
TAF, n (%)	2 (1.9%)	3 (1.0%)		2 (3.0%)	1 (1.5%)	
TDF, n (%)	3 (2.9%)	3 (1.0%)		3 (4.5%)	0	
adefovir, n (%)	0	2 (0.6%)		0	0	
Lamivudine, n (%)	1 (1.0%)	7 (2.2%)		1 (1.5%)	1 (1.5%)	
Anti-infective treatment, n (%)	101 (96.2%)	254 (80.9%)	<0.001	63 (94.0%)	63 (94.0%)	1.000
MELD score (mean ± SD)	22.01 ± 7.59	21.99 ± 8.03	0.986	22.87 ± 7.43	23.48 ± 8.53	0.659

ACLF, acute on chronic liver failure; ALF, acute liver failure; CLF, chronic liver failure; CRRT, continuous renal replacement therapy; ETV, entecavir; HAV, 5-HT3RA, 5-HT3 receptor antagonist; hepatitis A virus; HBV, hepatitis B virus; HCV, hepatitis C virus; HE, hepatic encephalopathy; HEV, hepatitis E virus; MELD score, Model For End-Stage Liver disease score; PA, plasma adsorption; PE, plasma exchange; SALF, subacute liver failure; SD, standard deviation; TAF, tenofovir alafenamide; TDF, tenofovir disoproxil fumarate.

Among all the patients, males made up a higher proportion (61.9% and 70.7% in the 5-HT3RA group and the control group, respectively). The age in the 5-HT3RA group and the control group had no statistical difference. In terms of etiology, hepatitis B virus (HBV) accounted for the largest proportion (58.1% and 55.1% in 5-HT3RA group and control group, respectively). ACLF accounted for the largest proportion (59% and 55.4% in 5-HT3RA group and control group, respectively). The proportion of diabetes mellitus patients in the control group was significantly higher than that in the 5-HT3RA group (10.8% vs. 2.9%, *p* = 0.013). The proportion of patients with nausea and vomiting symptoms (71.4% vs. 17.5%, *p* < 0.001), bleeding (40.0% vs. 23.6%, *p* = 0.001), secondary infection (86.7% vs. 72.6%, *p* = 0.003), hepatorenal syndrome (14.3% vs. 6.7%, *p* = 0.016), PE (50.5% vs. 33.1%, *p* = 0.001), CRRT (16.2% vs. 8.6%, *p* = 0.028), and anti-infective therapy (96.2% vs. 80.9%, *p* < 0.001) in the 5-HT3R group was significantly higher than that in the control group (Table 1). After PSM, the differences between the two groups were not statistically significant (Table 1). There was no significant difference in the baseline laboratory parameters between the two groups of patients (Supplementary Table S2).

### Characteristics of Patients With the Use of 5-HT3RAs According to Classifications of Liver Failure

According to the classifications of liver failure, patients with ALF, CLF, and ACLF had more males than females, except for patients with SALF. In patients with ALF, the main causes were severe infection (40%) and unknown reason (40%). The main cause of SALF was drug-induced (45.5%). The main cause of CLF and ACLF was HBV. Ondansetron accounted for the largest proportion of 5-HT3RAs used in all the patients (>70%). Among the patients with different liver failures, gender, etiology, the use of ondansetron, PE, CRRT, and antiviral therapy were statistically different (Supplementary Table S3).

### Clinical Outcomes

**FIGURE 1:**
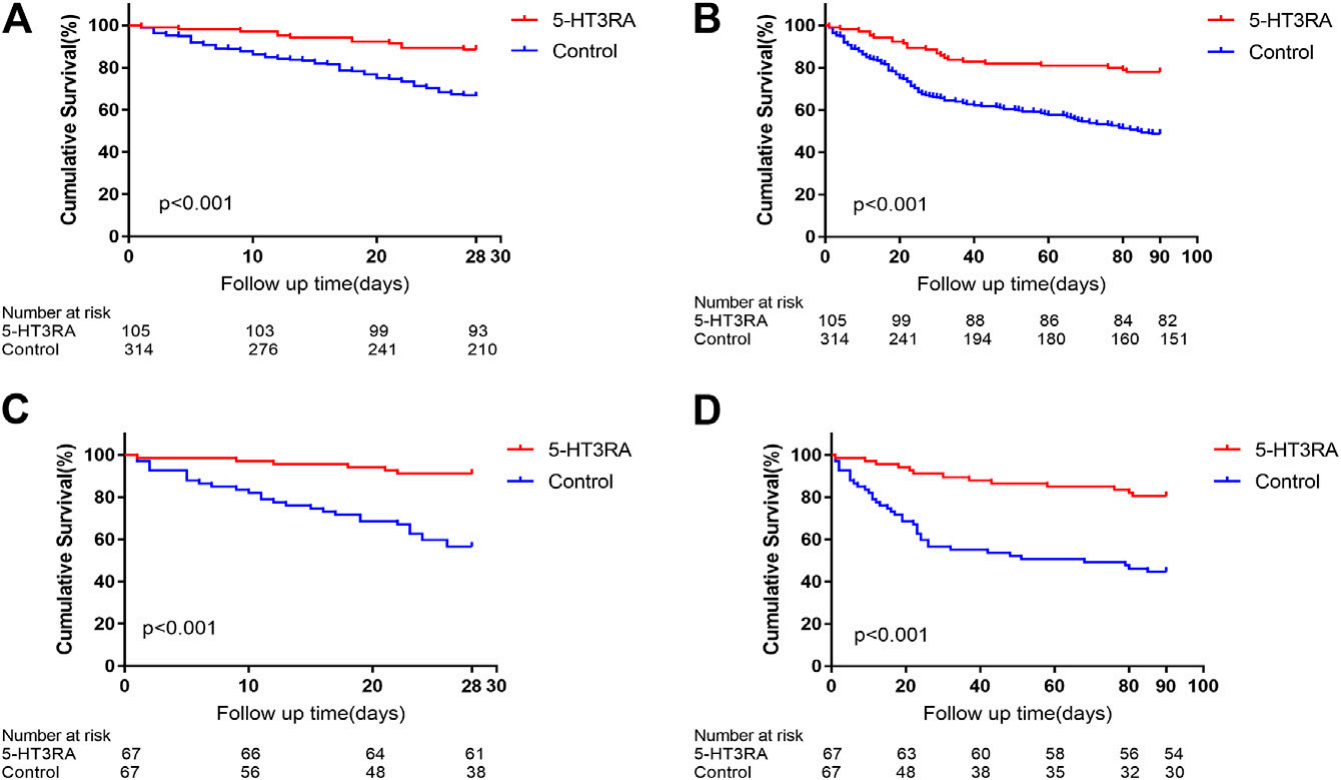
The 28 and 90 days Kaplan-Meier survival curves in patients with and without the use 5-HT3 receptor antagonists (5-HT3RA). **(A)** The 28 days survivals before propensity score matching (PSM). **(B)** The 90 days survivals before PSM. **(C)** The 28 days survivals after PSM. **(D)** The 90 days survivals after PSM.

Death/liver transplantation at 28 and 90 days occurred in 12 and 23 of 105 patients (11.4% and 21.9%, respectively) in 5-HT3RAs group and in 105 and 160 of 314 patients (33.4% and 51.0%, respectively) in control group (Figures 1A,B). Comparison of 28 days survivors and non-survivors showed that bleeding, HE, hepatorenal syndrome, electrolyte imbalance, encephaledema, the use of 5-HT3RAs, the times of 5-HT3RAs used, CRRT, antiviral therapy, anti-infection therapy, and MELD scores were significantly different (Supplementary Table S4). Comparison of 90 days survivors and non-survivors showed that nausea and vomiting symptoms, bleeding, ascites, secondary infection, HE, hepatorenal syndrome, electrolyte disturbances, use of 5-HT3RAs, the times of 5-HT3RAs used, CRRT, anti-infective treatment, and MELD scores were significantly different (Supplementary Table S5).

Cox regression analyses showed that HE, hepatopulmonary syndrome, the use of 5-HT3RAs, antiviral therapy, anti-infective treatment and MELD score were independent influencing factors for 28 days outcome. Notably, the use of 5-HT3RAs was significantly associated with decreased 28 days mortality (HR 0.18, 95% CI 0.10-0.34, *p* < 0.001, Table 2). HE, hepatopulmonary syndrome, the use of 5-HT3Rs, anti-infective therapy, MELD score were influencing factors for 90 days outcome. The use of 5-HT3RAs was also significantly associated with decreased 90 days mortality (HR 0.21, 95% CI 0.13-0.33, *p* < 0.001, Table 2).

**TABLE 2:** Univariate and multivariate Cox regression analyses of variables and 28 and 90 days outcomes before propensity score matching.

	Univariate		Multivariate	
	HR (95%CI)	*p* value	HR (95%CI)	*p* value
**28 days outcome**				
Bleeding	1.62 (1.11, 2.36)	0.012		
HE	4.01 (2.79, 5.78)	<0.001	2.69 (1.80, 4.000)	<0.001
Hepatorenal syndrome	2.49 (1.52, 4.07)	<0.001		
Hepatopulmonary syndrome	7.01 (1.73, 28.49)	0.006	12.42 (2.84, 54.37)	0.001
Electrolyte disturbance	1.53 (1.06, 2.20)	0.022		
Encephaledema	3.66 (1.96, 6.82)	<0.001		
Use of 5-HT3RAs	0.30 (0.17, 0.54)	<0.001	0.18 (0.10, 0.34)	<0.001
CRRT	3.21 (2.06, 5.00)	<0.001		
Antiviral therapy	0.68 (0.47, 0.98)	0.038	0.69 (0.48, 0.99)	0.046
Anti-infective treatment	2.08 (1.09, 3.98)	0.027	2.40 (1.20, 4.79)	0.013
MELD score	1.11 (1.09, 1.13)	<0.001	1.10 (1.08, 1.12)	<0.001
**90 days outcome**				
Nausea and vomiting symptoms	0.69 (0.49, 0.97)	0.032		
Bleeding	1.46 (1.07, 1.99)	0.017		
Ascites	1.58 (1.13, 2.20)	0.008		
HE	2.89 (2.15, 3.87)	<0.001	2.07 (1.50, 2.87)	<0.001
Hepatorenal syndrome	2.43 (1.60, 3.69)	<0.001		
Hepatopulmonary syndrome	7.01 (1.73, 28.49)	0.006	11.21 (2.63, 47.76)	0.001
Electrolyte disturbance	1.84 (1.38, 2.46)	<0.001		
Encephaledema	2.41 (1.31, 4.44)	0.005		
Use of 5-HT3RAs	0.35 (0.22, 0.54)	<0.001	0.21 (0.13, 0.33)	<0.001
5-HT3RA use ≥2 times	0.39 (0.19, 0.79)	0.009		
CRRT	2.42 (1.63, 3.61)	<0.001		
Anti-infective treatment	1.79 (1.11, 2.88)	0.017	1.65 (1.02, 2.68)	0.043
MELD score	1.09 (1.08, 1.11)	<0.001	1.09 (1.07, 1.11)	<0.001

CI, confidence interval; CRRT, continuous renal replacement therapy; HE, hepatic encephalopathy; HR, hazard ratio; 5-HT3RA, 5-HT3 receptor antagonist; MELD score, Model For End-Stage Liver disease score.

After PSM, death/liver transplantation at 28 and 90 days occurred in 6 and 13 of 67 patients (9.0% and 19.4%, respectively) in 5-HT3Rs group and in 29 and 37 of 67 patients (43.3% and 55.2%, respectively) in control group (Figures 1C,D). Cox regression analysis revealed that the use of 5-HT3RAs, CRRT and MELD score were independent influencing factors for 28 days outcome. 5-HT3RAs was significantly associated with decreased 28 days mortality (HR 0.10, 95%CI 0.04–0.26, *p* < 0.001, Table 3). HE, the use of 5-HT3RAs and MELD score were influencing factors for 90 days outcome. Among them, 5-HT3RAs was also significantly associated with decreased 90 days mortality (HR 0.16, 95%CI 0.08–0.31, *p* < 0.001, Table 3). In the unadjusted cohort, 5-HT3RAs was associated with reduced 28 days (HR 0.30, 95% CI 0.17-0.54, *p* < 0.001, Supplementary Table S6) and 90 days mortality (HR 0.35, 95% CI: 0.22-0.54, *p* < 0.001, Supplementary Table S6). After multivariate cox regression, PSM and IPTW, 5-HT3RAs remained significantly associated with reduced 28 and 90 days mortality (Supplementary Table S6).

**TABLE 3:** Univariate and multivariate Cox regression analyses of variables and 28 and 90 days outcomes after propensity score matching.

	Univariate		Multivariate	
	HR (95%CI)	*p* value	HR (95%CI)	*p* value
**28 days outcome**				
HE	3.25 (1.66, 6.35)	0.001		
Use of 5-HT3RAs	0.17 (0.70, 0.41)	<0.001	0.10 (0.04, 0.26)	<0.001
CRRT	3.44 (1.65, 7.19)	0.001	2.73 (1.21, 6.14)	0.015
MELD score	1.10 (1.06, 1.14)	<0.001	1.11 (1.06, 1.16)	<0.001
**90 days outcome**				
HE	2.81 (1.61, 4.89)	<0.001	1.95 (1.08, 3.54)	0.028
Hepatorenal syndrome	2.36 (1.18, 4.72)	0.015		
Use of 5-HT3RAs	0.26 (0.14, 0.49)	<0.001	0.16 (0.08, 0.31)	<0.001
CRRT	2.89 (1.50, 5.54)	0.001		
MELD score	1.09 (1.06, 1.13)	<0.001	1.11 (1.07, 1.15)	<0.001

CI, confidence interval; CRRT, continuous renal replacement therapy; HE, hepatic encephalopathy; HR, hazard ratio; 5-HT3RA, 5-HT3 receptor antagonist; MELD score, Model For End-Stage Liver disease score.

Before PSM, 5-HT3RA group had a significantly higher 28-days and 90-days survival rates than control group (28 days: 88.6% vs. 66.6%; 90 days: 78.1% vs. 48.8%, both *p* < 0.001, Figures 1A,B). After PSM, the 28-days and 90-days survivals of the 5-HT3RA group were still significantly higher than control group (28 days: 91.0% vs. 56.7%; 90 days: 80.6% vs. 44.8%, both *p* < 0.001, Figures 1C,D and Table 4).

**TABLE 4:** Comparison of 28 and 90 days survival for all patients and subgroup patients without (No) and with (Yes) the use of 5-HT3 receptor antagonists.

	Model		5-HT3RA	Survival (%)	Log-rank test *p* value
28 days outcome	All patients (*n* = 419)		No	66.6	<0.001
			Yes	88.6	
	PSM (*n* = 132)		No	56.7	<0.001
			Yes	91.0	
	Type of liver failure	ALF (*n* = 46)	No	63.9	0.117
			Yes	90	
		SALF (*n* = 93)	No	70.4	0.022
			Yes	95.5	
		CLF (*n* = 44)	No	72.7	0.064
			Yes	100	
		ACLF (*n* = 236)	No	64.4	0.005
			Yes	83.9	
	Etiology	HBV-related (*n* = 234)	No	67.1	0.003
			Yes	86.9	
		Drug-induced (*n* = 55)	No	66.7	0.088
			Yes	92.3	
		Alcoholic-induced (*n* = 25)	No	56.5	0.288
			Yes	100	
	Ondansetron (*n* = 401)		No	66.9	<0.001
			Yes	93.1	
	Ondansetron	ALF (*n* = 43)	No	63.9	0.078
			Yes	100	
		SALF (*n* = 91)	No	71.2	0.036
			Yes	95	
		CLF (*n* = 42)	No	72.7	0.093
			Yes	100	
		ACLF (*n* = 225)	No	64.4	<0.001
			Yes	90.2	
90 days outcome	All patients (*n* = 419)		No	48.8	<0.001
			Yes	78.1	
	PSM (*n* = 132)		No	44.8	<0.001
			Yes	80.6	
	Type of liver failure	ALF (*n* = 46)	No	58.3	0.074
			Yes	90	
		SALF (*n* = 93)	No	45.1	0.002
			Yes	86.4	
		CLF (*n* = 44)	No	54.5	0.090
			Yes	81.8	
		ACLF (*n* = 236)	No	47.6	0.001
			Yes	72.6	
	Etiology	HBV-related (*n* = 234)	No	50.1	0.001
			Yes	75.4	
		Drug-induced (*n* = 55)	No	52.4	0.055
			Yes	84.6	
		Alcoholic-induced (*n* = 25)	No	41.1	0.187
			Yes	100	
	Ondansetron (*n* = 401)		No	48.1	<0.001
			Yes	80.5	
	Ondansetron	ALF (*n* = 43)	No	58.3	0.054
			Yes	100	
		SALF (*n* = 91)	No	46.6	0.005
			Yes	85.0	
		CLF (*n* = 42)	No	54.5	0.168
			Yes	77.8	
		ACLF (*n* = 225)	No	47.1	<0.001
			Yes	76.5	

Abbreviations: ACLF, acute on chronic liver failure; ALF, acute liver failure; CLF, chronic liver failure; HBV, hepatitis B virus; 5-HT3RA, 5-HT3 receptor antagonist; SALF, subacute liver failure.

### Subgroup Analyses

**FIGURE 2:**
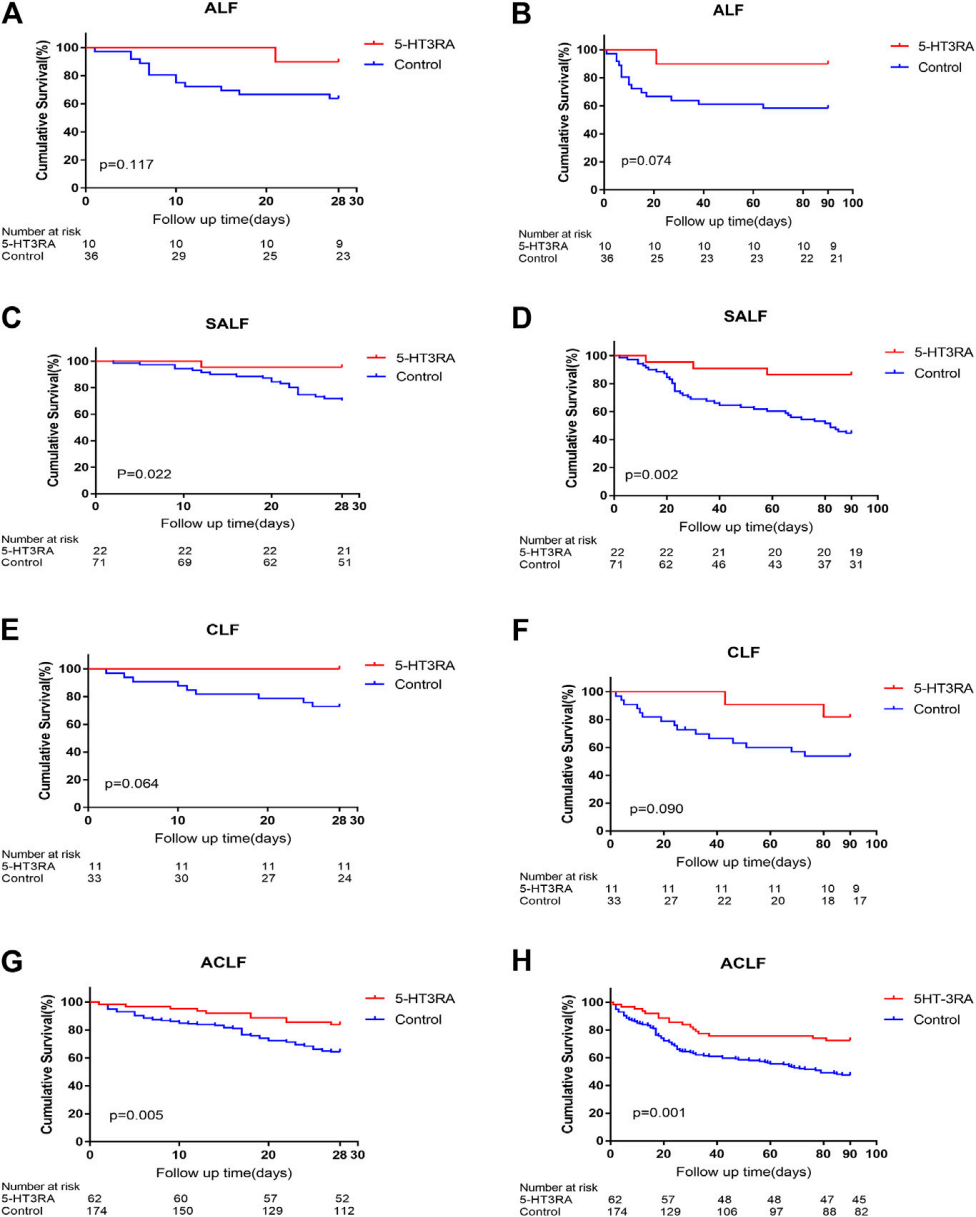
The 28 and 90 days Kaplan-Meier survival curves in different types of liver failure patients with or without the use of 5-HT3 receptor antagonists (5-HT3RA). **(A)** The 28 days survivals in acute liver failure (ALF). **(B)** The 90 days survivals in ALF. **(C)** The 28 days survivals in sub-acute liver failure (SALF). **(D)** The 90 days survivals in SALF. **(E)** The 28 days survivals in chronic liver failure (CLF). **(F)** The 90 days survivals in CLF. **(G)** The 28 days survivals in acute on chronic liver failure (ACLF). **(H)** The 90 days survivals in ACLF.

In ALF, the 28 and 90 days survivals in 5-HT3RA group were higher than those in the control group although there was no statistical difference (28 days: 90% vs. 63.9%, *p* = 0.117; 90 days: 90% vs. 58.3%, *p* = 0.074, Figures 2A,B). In SALF, the 28 and 90 days survivals of 5-HT3RA group was significantly higher than control group (28-days: 95.5% vs. 70.4%, *p* = 0.022; 90-days: 86.4% vs. 45.1%, *p* = 0.002, Figures 2C,D). In CLF, the 28 and 90 days survivals in 5-HT3RA group were higher than control group although there was no statistically significant difference (28 days: 100% vs. 72.7%, *p* = 0.064; 90 days: 81.8% vs. 54.5%, *p* = 0.090, Figures 2E,F). In ACLF, the 28 and 90 days survivals in 5-HT3RA group were significantly higher than control group (28 days: 83.9% vs. 64.4%, *p* = 0.005; 90 days: 72.6% vs. 47.6%, *p* = 0.001, Figures 2G,H and Table 4).

In HBV-associated liver failure, the 28 and 90 days survivals in 5-HT3RA group were significantly higher than those in control group (28 days: 86.9% vs. 67.1%, *p* = 0.003; 90 days: 75.4% vs. 50.1%, *p* = 0.001, Supplementary Figures S2A,B). In drug-induced liver failure, the 28 and 90 days survivals in 5-HT3RA group were higher than those in control group although there was no statistically significant difference (28 days: 92.3% vs. 66.7%, *p* = 0.088; 90 days: 84.6% vs. 52.4%, *p* = 0.055, respectively, Supplementary Figures S2C,D). In alcohol-related liver failure, the 28 and 90 days survivals in 5-HT3RA group were higher than those in control group with no statistically significant difference (28 days: 100% vs. 56.5%, *p* = 0.288; 90 days: 100% vs. 41.9%, *p* = 0.187, respectively, Supplementary Figures S2E,F, Table 4). Liver failure with other etiology was not analyzed because of the small number of patients.

In terms of different 5-HT3RA drugs, the 28 and 90 days survivals of patients who used ondansetron were significantly higher than those who did not (28 days: 93.1% vs. 66.9%, *p* < 0.001; 90 days: 80.5% vs. 48.1%, *p* < 0.0010, respectively, Supplementary Figures S3A,B, Table 4). The subgroup analysis of patients using ondansetron according to the classifications of liver failure resulted in similar results to the subgroup analysis in all the 5-HT3RAs (Supplementary Figures S3C–J). Subgroup analysis was not performed in other 5-HT3RAs because of the small number of patients.

## Discussion

This study is the first population-based study to establish an association between 5-HT3RAs and prognosis of patients with liver failure. In the patients included, there were more males than females. The etiologies of liver failure were varied and the 3 leading causes were HBV, alcohol, and drug. Drug was the main cause of SALF, accounting for 45.5%. There were 15.5% of the patients with indeterminate etiology. These results were similar to the report of southwest China (Xie et al., 2016), but different from the report of western countries (Sarin et al., 2019). ACLF was the predominant clinical type of liver failure, a finding similar to the report from other area of China (Liu et al., 2008).

This study showed that, in all patients and patients after PSM, HE, hepatopulmonary syndrome, the use of 5-HT3RAs, antiviral therapy and MELD score were independent influencing factors for 28 and 90 days outcome. It has been well known that the presence of complications such as HE and hepatopulmonary syndrome is a major risk factor for mortality in liver failure patients (Chen et al., 2019), antiviral treatment is effective in decreasing the short-term fatality of HBV-related ACLF (Zhang et al., 2014; Li et al., 2017), and the MELD score is widely used to predict the prognosis of liver failure (Katoonizadeh et al., 2007; Mishra and Rustgi, 2018). Most importantly, the use of 5-HT3RAs was associated with substantially reduced 28 and 90 days mortality in the patients with liver failure. This finding was consistently observed in unadjusted, multivariate-adjusted, propensity score-matched, and inverse probability treatment weighting analysis. The survival rate of liver failure patients with the use of 5-HT3RAs was significantly higher than that of patients without the use. Though, the difference of 28 and 90 days survivals in patients with ALF and CLF and in etiologies other than HBV was no statistically significant in subgroup analyses that may be related to the small numbers of patients in these subgroups. Our findings are consistent with previous studies in animal models showing that the 5-HT3RAs were able to reduce liver damage caused by various reasons including sepsis (Fiebich et al., 2004a), hemorrhagic shock (Liu et al., 2011), diabetes induced (Gholizadeh-Ghaleh Aziz et al., 2019; Amini et al., 2020) and obesity-associated (Haub et al., 2011) liver injuries.

The mechanisms pertinent to the association of 5-HT3RAs with a significantly reduced risk of mortality in liver failure patients might be multiple and complex. First, 5-HT3RAs have anti-oxidative, anti-inflammatory and immunomodulatory properties (Fiebich et al., 2004a; Fiebich et al., 2004b; Stratz et al., 2012; Gong et al., 2019). Experiments in animals have indicated that 5-HT3RAs can alleviate liver damage induced by various causes via antioxidant and anti-inflammatory effects (Fiebich et al., 2004a; Haub et al., 2011; Liu et al., 2011; Gholizadeh-Ghaleh Aziz et al., 2019; Amini et al., 2020). Host immune dysregulations including T cell responses are considerably involved in the development of liver failure (Khamri et al., 2017) while 5-HT3RA tropisetron was found to suppress T cell activation (Vega et al., 2005). Therefore, the immunoregulatory action of 5-HT3RAs may also contribute to the effect on liver failure. Second, intestinal motility and permeability and gut microbiota play important role in hepatic disorders (Bajaj et al., 2014; Chopyk and Grakoui, 2020). 5-HT3R is a key factor in the regulation of intestinal motility and permeability. 5-HT3RAs such as tropisetron can reduce hepatic lesions of obesity-associated fatty liver disease in mice by reducing portal vein plasma endotoxin levels, attenuating the increased MyD88 and tumor necrosis factor-α mRNA expression in the liver, and increasing tight junction proteins in the duodenum (Haub et al., 2011). Gut microbiota can regulate 5-HT3R expression and modulate host secretory response to 5-HT (Bhattarai et al., 2017). In sepsis-induced liver injury mice, granisetron, a 5-HT3RA, generated by intestinal microbiota confers resistance to sepsis, protects mice against death and liver injury, and reduces proinflammatory cytokine expression by macrophages after lipopolysaccharide challenge (Fiebich et al., 2004a). In septic patients, gut microbial granisetron levels were negatively correlated with plasma transaminase levels (Fiebich et al., 2004b). Therefore, 5-HT3RAs may exert hepatoprotective effect on liver failure by the regulation of intestinal motility and permeability and the involvement of gut microbiota. Third, inflammation-associated mitochondrial dysfunction is revealed to be a potential mechanism of organ failures in ACLF (Moreau et al., 2020). 5-HT3RA tropisetron is shown to alleviate the mitochondrial dysfunction of the cerebral cortex in mice following social isolation stress (Haj-Mirzaian et al., 2016). Thus, the mitochondrial dysfunction-attenuating effect of 5-HT3RAs may potentially play a role in alleviating liver failure. Fourth, multiple organ failure is directly related to the mortality of liver failure patients (Arroyo et al., 2015; Mahmud et al., 2020). 5-HT3RAs have multiorgan protective effects. For example, 5-HT3RA tropisetron shows renal protective effect on early diabetic nephropathy (Barzegar-Fallah et al., 2015) and improves pancreas function, increases insulin synthesis and secretion, and attenuates pancreas apoptosis in streptozotocin-induced diabetic rats (Naderi et al., 2020a; Naderi et al., 2020b). 5-HT3RAs, ondansetron, tropisetron and palonosetron, show anti-inflammatory effect, restore the delayed gastrointestinal transit and exhibit therapeutic action against post-operative ileus (Maehara et al., 2015). In mice, ondansetron shows potential therapeutic effect for Alzheimer's disease (Shinohara et al., 2019) and tropisetron shows protective action against brain aging (Mirshafa et al., 2020). These findings suggest that the multiple organ protective effect of 5-HT3RAs may potentially play a paramount role in reducing the mortality of liver failure. Fifth, thrombotic disorders including portal vein thrombosis and intrahepatic microthrombosis may be involved in the pathogenesis of liver disease including liver failure (Pieri et al., 2013). A recent study demonstrated that ondansetron considerably reduces the risk of hospital-acquired venous thromboembolism (Datta et al., 2020). Therefore, 5-HT3RAs such as ondansetron may also exert a therapeutic effect on liver failure through reduction of thrombotic disorders.

Of note, the doses of 5-HT3RAs used in the patients of this study were very small and this may call into question of the therapeutic effect on liver failure. The 5-HT3RAs used such as ondansetron are highly selective and preclinical studies in rat have shown the effects of small doses of ondansetron on cognition, behavioural sensitisation, and epilepsy (Kwan et al., 2020). In a recent study, perioperative use of 5-HT3RAs (palonosetron or ramosetron) is associated with increased recurrence-free survival in patients after thoracotomy for lung cancer although the doses used were not presented in detail and ondansetron was excluded from the 5-HT3RAs (Lee et al., 2019). It is suggested that small doses of 5-HT3RAs may also exhibit peripheral effect. Furthermore, we did not observe a dose-dependent effect of the 5-HT3RAs on liver failure. Therefore, the optimal dose and timing of 5-HT3RAs need to be determined in future studies.

Lastly and importantly, there are many potential limitations or confounders that may influence the results of our study. First, this study is a retrospective study, and there may have been some confounding factors and clinical variables that we were unable to measure and control for. Though, we adopted a variety of strategies such as multivariate adjustment, PSM and IPTW to reduce the influence of data bias and confounding variables on the outcome. Second, single center and small sample size of the study may also compromise the potency of our analyses, especially the subgroup analyses. It is difficult to obtain large samples of patient data at present owing to the unconventional use of 5-HT3RAs for liver failure. Third, the etiology of liver failure in this study was mainly HBV and thus the findings might not be applicable to liver failure mainly related to other reasons although we included patients with liver failure associated with etiologies other than HBV in the analysis. Fourth, this study was unable to include a validation analysis due to the lack of other eligible patient populations. Therefore, additional studies are definitely needed to verify the findings of this study.

## Conclusion

This study suggests that the use of 5-HT3RAs is associated with significantly increased short-term survivals of patients with liver failure, especially SALF and ACLF. Due to the lack of effective medical treatment, the poor prognosis of liver failure and the clinical safety profile of 5-HT3RAs in humans, our findings might have considerable clinical implications for the treatment of liver failure. High quality multi-center, large-sample, prospective, randomized and double-blind controlled studies are required to confirm our findings.

## Data Availability

The original contributions presented in the study are included in the article/Supplementary Material, further inquiries can be directed to the corresponding author.

## Abbreviations

ACLF, acute on chronic liver failure; ALB, albumin; ALF, acute liver failure; CI, confidence interval; CLF, chronic liver failure; CRRT, continuous renal replacement therapy; HBV, hepatitis B virus; HE, hepatic encephalopathy; HR, hazard ratio; 5-HT3RAs, 5-hydroxytryptamine 3 receptor antagonists; INR, international normalized ratio; IPTW, inverse probability treatment weighting; LT, liver transplantation; MELD, model for end-stage liver disease; OR, odds ratio ; PA, plasma adsorption; PE, plasma exchange; PSM, propensity score matching; PT, prothrombin time; PTA, prothrombin activity; SALF, sub-acute liver failure; TAF, tenofovir alafenamide; TBIL, total bilirubin; TDF, tenofovir disoproxil; ULN, upper limit of normal; VIF, variance inflation factor.
