# Supplementary material for: Association of 5-Hydroxytryptamine 3 Receptor Antagonists With the Prognosis of Liver Failure
Source: Front Pharmacol. 2021 Apr 22;12:648736. doi: 10.3389/fphar.2021.648736 (PMC8100675; doi:10.3389/fphar.2021.648736)
Supplement: Supplementary file 1 [file datasheet1.doc]

**Supplementary Material**

509 patients were diagnosed with liver failure in the First Affiliated Hospital of Xi'an Jiaotong University from May, 2013 to June, 2019

Excluded：

Age＜18 years (n=5)

Pregnant (n=1)

With malignant tumor (n=54)

With a history of liver transplantation (n=2)

With circulatory failure (n=2)

With incomplete data (n=3)

442 patients eligible for study

Excluded:

Lost to follow-up (n=23)

419 patients included in final analysis

5-HT3 receptor antagonist group

(n=105)

Control group

(n=314)

**Supplementary Figure S1. Flow chart of the study population.**

**
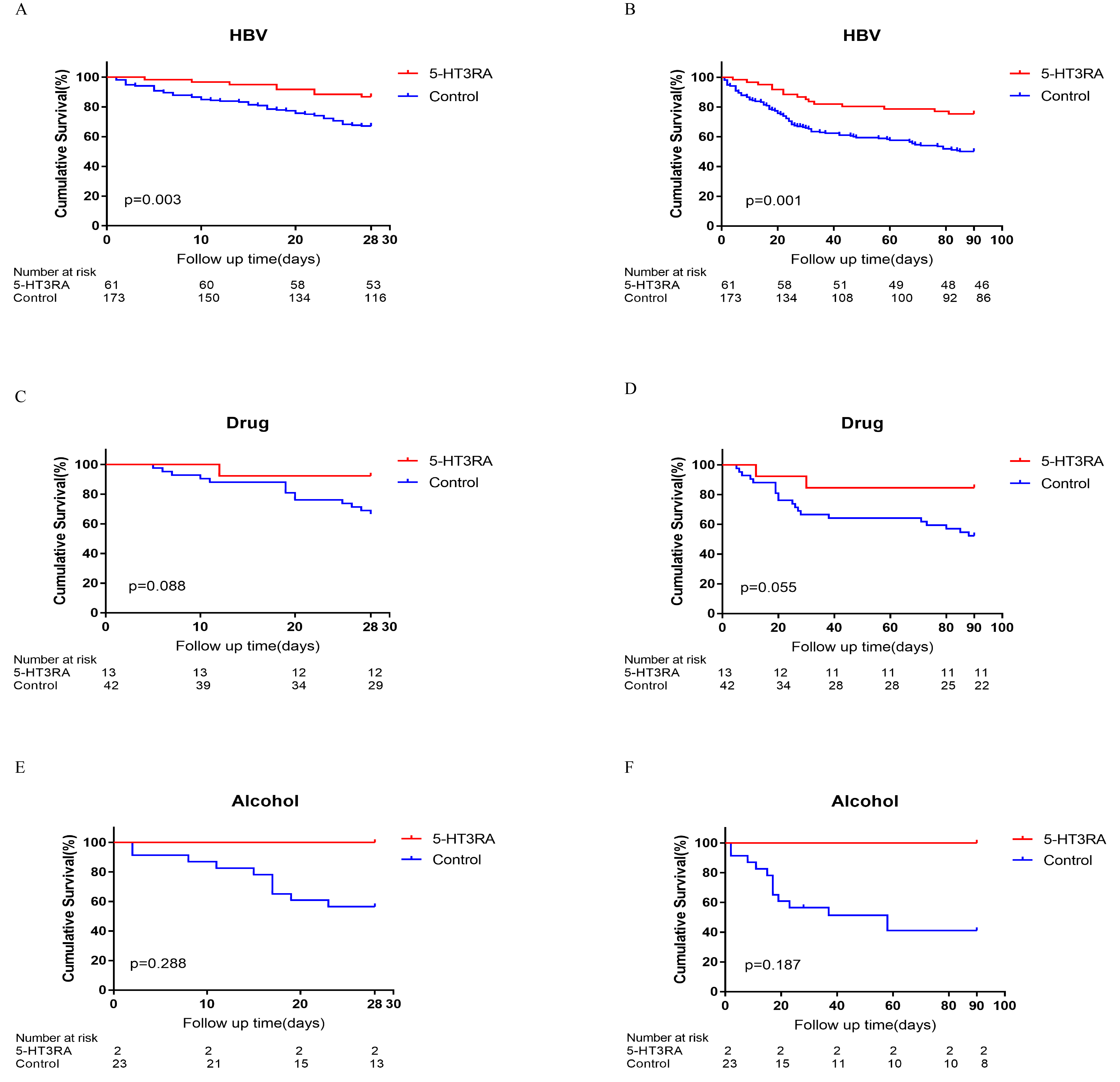
**

**Supplementary Figure S2. The 28-day and 90-day Kaplan-Meier survival curves in different etiology (include HBV-associated liver failure,drug-induced liver failure and alcohol-related liver failure) of Liver failure** **patients with or without the use of 5-HT3 receptor antagonists (5-HT3RA).** (A) The 28-day survivals in HBV-associated liver failure. (B) The 90-day survivals in HBV-associated liver failure. (C) The 28-day survivals in drug-induced liver failure. (D) The 90-day survivals in drug-induced liver failure. (E) The 28-day survivals in alcohol-related liver failure. (F) The 90-day survivals in alcohol-related liver failure.

**
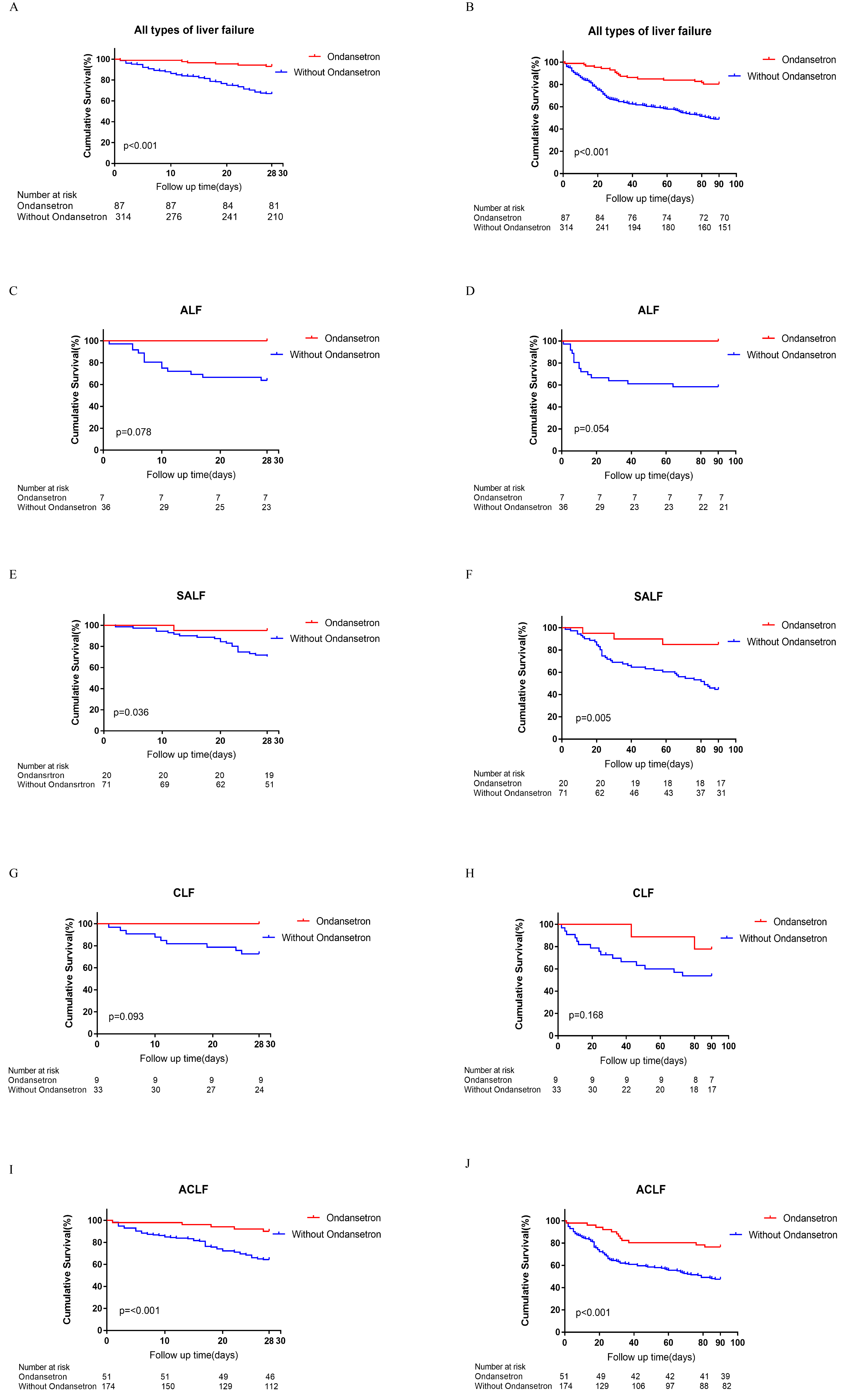
**

**Supplementary Figure S3. The 28-day and 90-day Kaplan-Meier survival curves in all types of liver failure and different types of liver failure patients with or without the use of ondansetron.** (A) The 28-day survivals in all types of liver failure. (B) The 90-day survivals in all types of liver failure. (C) The 28-day survivals in acute liver failure (ALF). (D) The 90-day survivals in ALF. (E) The 28-day survivals in sub-acute liver failure (SALF). (F) The 90-day survivals in SALF. (G) The 28-day survivals in chronic liver failure (CLF). (H) The 90-day survivals in CLF. (I) The 28-day survivals in acute on chronic liver failure (ACLF). (J) The 90-day survivals in ACLF.

**Supplementary Table S1. Baseline characteristics after inverse probability treatment weighting (IPTW).**

|  | 5-HT3RA group | Control group | STD | *p* value |
| --- | --- | --- | --- | --- |
| Gender |  |  |  | 0.003 |
| Male,% | 48.6% | 65.1% | 0.338 |  |
| Female,% | 51.4% | 34.9% | 0.338 |  |
| Age (years) | 46.37±13.59 | 46.86±13.16 | 0.037 | 0.746 |
| Ethnic |  |  |  |  |
| Han | 100% | 98.8% | 0.156 | 0.271 |
| Non-Han |  | 1.2% |  |  |
| Etiology |  |  |  | 0.826 |
| HAV,% | 0.3% | 0 | - |  |
| HBV,% | 62.6% | 53.5% | 0.184 |  |
| HCV,% | 0 | 1% | - |  |
| HEV,% | 0 | 0.7% | - |  |
| Drug-induced, % | 10.4% | 13.6% | 0.097 |  |
| Alcoholic, % | 5.7% | 5.7% | 0.003 |  |
| Acute mushroom poisoning,% | 0 | 0.2% | - |  |
| Severe infection,% | 3.4% | 4.9% | 0.075 |  |
| Autoimmune liver disease,% | 4.5% | 2.5% | 0.110 |  |
| Cholestatic liver disease,% | 1.1% | 2.2% | 0.080 |  |
| Hepatolenticular degeneration,% | 0 | 0.5% | - |  |
| Budd-Chiari syndromen,% | 0 | 0.2% | - |  |
| Unknown reason,% | 12% | 15% | 0.086 |  |
| Classification of liver failure |  |  |  | 0.024 |
| ALF,% | 12.9% | 10.9% | 0.062 |  |
| SALF,% | 14.1% | 22.3% | 0.214 |  |
| CLF,% | 5.2% | 12.9% | 0.269 |  |
| ACLF,% | 67.8% | 53.9% | 0.287 |  |
| Hypertension, % | 6.5% | 8.3% | 0.069 | 0.556 |
| Diabetes mellitus, % | 4% | 8.9% | 0.198 | 0.113 |
| Smoking status, % | 29.9% | 39.2% | 0.198 | 0.091 |
| Drinking habit, % | 26.6% | 30.6% | 0.089 | 0.444 |
| Nausea and vomiting symptoms, % | 32.1% | 31.0% | 0.022 | 0.844 |
| Complication |  |  |  |  |
| Bleeding, % | 27.4% | 25.6% | 0.041 | 0.720 |
| Ascites, % | 55.4% | 64.7% | 0.190 | 0.095 |
| Secondary infection, % | 71.7% | 76.6% | 0.112 | 0.322 |
| HE, % | 28.6% | 29.3% | 0.015 | 0.895 |
| Hepatorenal syndrome, % | 6.3% | 7.0% | 0.029 | 0.805 |
| Hepatopulmonary syndrome, % | 0.3% | 0.3% | 0.003 | 0.978 |
| Electrolyte disturbance, % | 37.6% | 36.6% | 0.020 | 0.859 |
| Encephaledema,% | 2.1% | 4% | 0.111 | 0.366 |
| Artificial liver support treatment |  |  |  |  |
| PE, % | 33.5% | 36.5% | 0.063 | 0.587 |
| PA, % | 3.7% | 8.6% | 0.206 | 0.101 |
| CRRT, % | 13.9% | 9.1% | 0.148 | 0.174 |
| Antiviral therapy, % | 62.2% | 52.3% | 0.200 | 0.084 |
| Antiviral drugs |  |  |  | 0.528 |
| ETV, % | 54.5% | 44.5% | 0.201 |  |
| TAF, % | 0.7% | 0.9% | 0.022 |  |
| TDF, % | 1.5% | 1.1% | 0.032 |  |
| Adefovir,% | 0 | 0.5% | - |  |
| Lamivudine,% | 2.7% | 1.9% | 0.050 |  |
| Anti-infective treatment, % | 79.6% | 84.6% | 0.132 | 0.237 |
| MELD score (mean±SD) | 21.16±7.27 | 22.26±8.07 | 0.144 | 0.190 |

Abbreviations: ACLF, acute on chronic liver failure; ALF, acute liver failure; CLF, chronic liver failure; CRRT, continuous renal replacement therapy; ETV, entecavir; HAV, hepatitis A virus; HBV, hepatitis B virus; HCV, hepatitis C virus; HE, hepatic encephalopathy; HEV, hepatitis E virus; 5-HT3RA, 5-HT3 receptor antagonist; MELD score, model for end-stage liver disease score; PA, plasma adsorption; PE, plasma exchange; SALF, subacute liver failure; SD, standard deviation; STD, standardized difference; TAF, tenofovir alafenamide; TDF, tenofovir disoproxil fumarate.

Supplementary Table S2. Baseline laboratory characteristic between patients with and without the use of 5-HT3 receptor antagonists.

| **Variable** | 5-HT3RA group (n=105) | Control group  (n=309) | *p* value |
| --- | --- | --- | --- |
| AST(IU/L) | 161(18,7770) | 189(16,7097) | 0.414 |
| ALT(IU/L) | 181(10,6550) | 192.25(3.68,5775) | 0.907 |
| ALP(IU/L) | 161(50.3,1202) | 152.5(41,1829.29) | 0.875 |
| GGT(IU/L) | 86(10.10,89137) | 87.5(7,1693.1) | 0.745 |
| TBIL(μmol/L) | 298.5(46.3,676.60) | 281.2(26.3,822.4) | 0.551 |
| CHOL(mmol/L) | 2.16(0.83,14.8) | 2.31(0.9,5.94) | 0.366 |
| TP (g/L) | 60.41±11.91 | 62.64±10.01 | 0.086 |
| ALB (g/L) | 31.69±5.76 | 31.88±5.56 | 0.765 |
| BUN(mmol/L) | 5.16(0.8,33.56) | 4.93(0.7,42.82) | 0.345 |
| CRE(μmol/L) | 58.5(28,258) | 61(25.88,598) | 0.802 |
| Cys-C(mg/L) | 1.15(0.45,3.21) | 1.13(0.9,7.38) | 0.370 |
| serum potassium,(mmol/L) | 3.78(2.34,5.97) | 3.73(2.16,7.21) | 0.303 |
| serum sodium(mmol/L) | 136(120，152) | 137.8(116,149) | 0.084 |
| RBC (×10^12^/L) | 3.74±0.95 | 3.72±0.89 | 0.896 |
| HGB (g/L) | 116.10±27.97 | 117.82±25.24 | 0.555 |
| PLT (×10^9^/L) | 104(3，375) | 93(5,655) | 0.445 |
| WBC (×10^9^/L) | 6.23(0.63,29.24) | 6.00(1.35,76.04) | 0.107 |
| LYMPH (×10^9^/L) | 0.95(0.11,5.12) | 0.98(0.07,3.67) | 0.842 |
| NEUT (×10^9^/L) | 4.71(0.31,26.87) | 4.1(0.54,72.47) | 0.079 |
| PT (s) | 21.5(12.4,50.7) | 22.2(12.4,120) | 0.554 |
| PTA (%) | 38.33(11.5,103.6) | 36.2(7,124) | 0.101 |
| INR | 1.87(0.94,5.56) | 1.96(0.94,13.02) | 0.506 |
| AFP (ng/mL) | 29.37(0.605,1173) | 46.76(0.695,3355) | 0.697 |

Abbreviations: AFP, alpha-fetoprotein; ALB, albumin; ALP, alkaline phosphatase; ALT, alanine aminotransferase; AST, aspartate aminotransferase; BUN, blood urea nitrogen; CHOL, cholesterol; CRE, creatinine; Cys-C, cystatin C; GGT, gamma-glutamine transpeptidase; HGB, hemoglobin; 5-HT3RA, 5-HT3 receptor antagonist; INR, international normalized ratio; LYMPH, lymphocyte count; NEUT, neutrophil count; PLT, platelet count; PT, prothrombin time; PTA, prothrombin activity; RBC, red blood cell; TBIL, total bilirubin; TP, total protein; WBC, white blood cell.

Supplementary Table S3. Baseline characteristics of patients with the use of 5-HT3 receptor antagonists according to classifications of liver failure (n=105).

| **Variable** | ALF  (n=10) | SALF  (n=22) | CLF  (n=11) | ACLF  (n=62) | *p* value |
| --- | --- | --- | --- | --- | --- |
| Gender |  |  |  |  | 0.004 |
| Male, n(%) | 5(50%) | 7(31.8%) | 7(63.6%) | 46(74.2%) |  |
| Female, n(%) | 5(50%) | 15(68.2%) | 4(36.4%) | 16(25.8%) |  |
| Age (mean±SD) | 49.2±15.59 | 48.32±14.26 | 57.73±16.16 | 45.29±13.69 | 0.067 |
| Etiology |  |  |  |  | ＜0.001 |
| HAV, n(%) | 0 | 1(4.5%) | 0 | 0 |  |
| HBV, n(%) | 0 | 5(22.7%) | 8(72.7%) | 48(77.4%) |  |
| Drug-induced, n(%) | 1(10%) | 10(45.5%) | 1(9.1%) | 1(1.6%) |  |
| Alcoholic, n(%) | 0 | 0 | 0 | 2(3.2%) |  |
| Severe infection-associated, n(%) | 4(40%) | 1(4.5%) | 0 | 1(1.6%) |  |
| Autoimmune liver disease n(%) | 1(10%) | 0 | 0 | 1(1.6%) |  |
| Cholestatic liver disease, n(%) | 0 | 0 | 1(9.1%) | 1(1.6%) |  |
| Unknown reason, n(%) | 4(40%) | 5(22.7%) | 1(9.1%) | 8(12.9%) |  |
| Hypertension, n(%) | 0 | 4(18.2%) | 2(18.2%) | 6(9.7%) | 0.400 |
| Diabetes mellitus, n(%) | 0 | 0 | 0 | 3(4.8%) | 0.778 |
| Smoking status, n(%) | 3(30%) | 4(18.2%) | 5(45.5%) | 25(40.3%) | 0.245 |
| Drinking habit, n(%) | 3(30%) | 4(18.2%) | 3(27.3%) | 18(29%) | 0.783 |
| Nausea and vomiting symptoms, n(%) | 6(60%) | 19(86.4%) | 9(81.8%) | 41(66.1%) | 0.217 |
| Complication |  |  |  |  |  |
| Bleeding, n(%) | 4(40%) | 6(27.3%) | 4(36.4%) | 18(45.2%) | 0.533 |
| Ascites, n(%) | 7(70%) | 13(59.1%) | 10(90.9%) | 45(72.6%) | 0.294 |
| Secondary infection, n(%) | 10(100%) | 19(86.4%) | 9(81.8%) | 53(85.5%) | 0.688 |
| HE, n(%) | 5(50%) | 3(13.6%) | 2(18.2%) | 24(38.7%) | 0.068 |
| Hepatorenal syndrome, n(%) | 0 | 1(4.5%) | 1(9.1%) | 13(21.0%) | 0.153 |
| Hepatopulmonary syndrome, n(%) | 0 | 0 | 0 | 1(16%) | 1.000 |
| Electrolyte disturbance, n(%) | 7(70%) | 7(31.8%) | 5(45.5%) | 23(37.1%) | 0.203 |
| Encephaledema, n(%) | 1(10%) | 2(9.1%) | 0 | 1(1.6%) | 0.161 |
| Times of 5-HT3RAs used |  |  |  |  | 0.572 |
| ＜2 times, n(%) | 7(70%) | 16(72.7%) | 8(72.7%) | 36(58.1%) |  |
| ≥2 times, n(%) | 3(30%) | 6(27.3%) | 3(27.3%) | 26(41.9%) |  |
| 5-HT3RAs |  |  |  |  |  |
| Ondansetron, n(%) | 7(70%) | 20(90.9%) | 10(90.9%) | 61(98.4%) | 0.010 |
| Granisetron, n(%) | 1(10%) | 1(4.5%) | 1(9.1%) | 7(11.3%) | 0.879 |
| Palonosetron, n(%) | 0 | 0 | 1(9.1%) | 2(3.2%) | 0.576 |
| Tropisetron, n(%) | 1(10%) | 1(4.5%) | 0 | 2(3.2%) | 0.522 |
| Artificial liver support treatment |  |  |  |  |  |
| PE, n(%) | 7(70%) | 15(68.2%) | 2(18.2%) | 29(46.8%) | 0.027 |
| PA, n(%) | 1(10%) | 2(9.1%) | 2(18.2%) | 3(4.8%) | 0.261 |
| CRRT, n(%) | 5(50%) | 2(9.1%) | 0 | 10(16.1%) | 0.018 |
| Antiviral therapy, n(%) | 3(30%) | 6(27.3%) | 5(45.5%) | 47(75.8%) | ＜0.001 |
| Antiviral drugs |  |  |  |  | ＜0.001 |
| ETV, n(%) | 0 | 3(13.6%) | 5(45.5%) | 40(64.5%) |  |
| TAF, n(%) | 0 | 1(4.5%) | 0 | 1(1.6%) |  |
| TDF, n(%) | 0 | 0 | 0 | 3(4.8%) |  |
| Adefovir, n(%) | 0 | 0 | 0 | 0 |  |
| Lamivudine,n(%) | 0 | 0 | 0 | 1(1.6%) |  |
| Anti-infective treatment | 10(100%) | 20(90.9%) | 11(100%) | 60(96.8%) | 0.614 |
| MELD score | 24.20±5.25 | 19.95±7.07 | 17.91±6.24 | 23.11±7.99 | 0.071 |

Abbreviations: ACLF, acute on chronic liver failure; ALF, acute liver failure; CLF, chronic liver failure; CRRT, continuous renal replacement therapy; ETV, entecavir; HAV, hepatitis A virus; HBV, hepatitis B virus; HCV, hepatitis C virus; HE, hepatic encephalopathy; HEV, hepatitis E virus; 5-HT3RA, 5-HT3 receptor antagonist; MELD score, model for end-stage liver disease score; PA, plasma adsorption; PE, plasma exchange; SALF, subacute liver failure; SD, standard deviation; TAF, tenofovir alafenamide; TDF, tenofovir disoproxil fumarate.

Supplementary Table S4. Characteristics of patients between 28-day survivors and non-survivors (death or liver transplantation).

| **Variable** | Survivors (n=302) | Non-survivors (n=117) | *p* value |
| --- | --- | --- | --- |
| Gender |  |  | 0.974 |
| Male, n(%) | 207(68.5%) | 80(68.4%) |  |
| Female,n(%) | 95(31.5%) | 37(31.6%) |  |
| Age (years) | 47.01±13.71 | 47.23±13.21 | 0.931 |
| Ethnic |  |  | 0.328 |
| Han | 297(98.3%) | 117(100%) |  |
| Non-Han | 5(1.7%) | 0 |  |
| Etiology |  |  | 0.434 |
| HAV, n(%) | 1(0.3%) | 0 |  |
| HBV, n(%) | 169(56.0%) | 65(55.6%) |  |
| HCV, n(%) | 2(0.7%) | 2(1.7%) |  |
| HEV, n(%) | 3(1.0%) | 0 |  |
| Drug-induced, n(%) | 40(13.2%) | 15(12.8%) |  |
| Alcoholic, n(%) | 15(5.0%) | 10(8.5%) |  |
| Acute mushroom poisoning, n(%) | 1(0.3%) | 0 |  |
| Severe infection, n(%) | 8(2.6%) | 1(0.9%) |  |
| Autoimmune liver disease, n(%) | 9(3.0%) | 1(0.9%) |  |
| Cholestatic liver disease, n(%) | 7(2.3%) | 2(1.7%) |  |
| Hepatolenticular degeneration | 0 | 2(1.7%) |  |
| Budd-Chiari syndromen, n(%) | 1(0.3%) | 0 |  |
| unknown reason, n(%) | 46(15.2%) | 19(16.2%) |  |
| Classification of liver failure |  |  | 0.393 |
| ALF, n(%) | 32(10.6%) | 14(12.0%) |  |
| SALF, n(%) | 71(23.5%) | 22(18.8%) |  |
| CLF, n(%) | 35(11.6%) | 9(7.7%) |  |
| ACLF, n(%) | 164(54.3%) | 72(61.5%) |  |
| Hypertension, n(%) | 25(8.3%) | 7(6%) | 0.427 |
| Diabetes mellitus, n(%) | 23(7.6%) | 14(12%) | 0.159 |
| Smoking status, n(%) | 121(40.1%) | 52(44.4%) | 0.414 |
| Alcohol drinking, n(%) | 94(31.1%) | 41(35%) | 0.441 |
| Nausea and vomiting symptoms | 96(31.8%) | 34(29.1%) | 0.588 |
| Complication |  |  |  |
| Bleeding, n(%) | 74(24.5%) | 42(35.9%) | 0.019 |
| Ascites, n(%) | 198(65.6%) | 82(70.1%) | 0.378 |
| Secondary infection, n(%) | 224(74.2%) | 95(81.2%) | 0.130 |
| HE, n(%) | 58(19.2%) | 63(53.8%) | ＜0.001 |
| Hepatorenal syndrome, n(%) | 17(5.6%) | 19(16.2%) | 0.001 |
| Hepatopulmonary syndrome, n(%) | 0 | 2(1.7%) | 0.077 |
| Electrolyte disturbance, n(%) | 105(34.8%) | 55(47%) | 0.021 |
| Encephaledema, n(%) | 6(2.0%) | 11(9.4%) | 0.001 |
| Use of 5-HT3RAs | 93(30.8%) | 12(10.3%) | ＜0.001 |
| Times of 5-HT3RAs used |  |  | 0.033 |
| ＜2 times, n(%) | 269(89.1%) | 112(95.7%) |  |
| ≥2 times, n(%) | 33(10.9%) | 5(4.3%) |  |
| Artificial liver support treatment |  |  |  |
| PE, n(%) | 110(36.4%) | 47(40.2%) | 0.447 |
| PA, n(%) | 28(9.3%) | 12(10.3%) | 0.758 |
| CRRT, n(%) | 19(6.3%) | 25(21.4%) | ＜0.001 |
| Antiviral therapy,n(%) | 173(57.3%) | 55(47%) | 0.058 |
| Antiviral drugs |  |  | 0.481 |
| ETV, n(%) | 142(47.0%) | 50(42.7%) |  |
| TAF, n(%) | 4(1.3%) | 1(0.9%) |  |
| TDF, n(%) | 6(2.0%) | 0 |  |
| Adefovir,n(%) | 1(0.3%) | 1(0.9%) |  |
| lamivudine,n(%) | 5(1.7%) | 3(2.6%) |  |
| Anti-infective treatment | 248(82.1%) | 107(91.5%) | 0.017 |
| MELD score | 19.67±6.25 | 28±8.61 | ＜0.001 |

Abbreviations: ACLF, acute on chronic liver failure; ALF, acute liver failure; CLF, chronic liver failure; CRRT, continuous renal replacement therapy; ETV, entecavir; HAV, hepatitis A virus; HBV, hepatitis B virus; HCV, hepatitis C virus; HE, hepatic encephalopathy; HEV, hepatitis E virus; 5-HT3RA, 5-HT3 receptor antagonist; MELD score, model for end-stage liver disease score; PA, plasma adsorption; PE, plasma exchange; SALF, subacute liver failure; SD, standard deviation; TAF, tenofovir alafenamide; TDF, tenofovir disoproxil fumarate.

Supplementary Table S5. Characteristics of 90-day survivors and non-survivors (death or liver transplantation).

| **Variable** | Survivors (n=236) | Non-survivors (n=183) | *p* value |
| --- | --- | --- | --- |
| Gender |  |  | 0.890 |
| Male, n(%) | 161(68.2%) | 126(68.9%) |  |
| Female, n(%) | 75(31.8%) | 57(31.1%) |  |
| Age | 46.46±13.64 | 48.01±13.44 | 0.247 |
| Ethnic |  |  |  |
| Han | 231(97.9%) | 183(100%) | 0.071 |
| Non-Han | 5(2.1%) | 0 |  |
| Etiology |  |  | 0.788 |
| HAV, n(%) | 1(0.4%) | 0 |  |
| HBV, n(%) | 133(56.4%) | 101(55.2%) |  |
| HCV, n(%) | 1(0.4%) | 3(1.6%) |  |
| HEV, n(%) | 2(0.9%) | 1(0.5%) |  |
| Drug-induced, n(%) | 33(14.0%) | 22(12%) |  |
| Alcoholic, n(%) | 12(5.1%) | 13(7.1%) |  |
| Acute mushroom poisoning, n(%) | 1(0.4%) | 0 |  |
| Severe infection, n(%) | 6(2.5%) | 3(1.6%) |  |
| Autoimmune liver disease, n(%) | 6(2.5%) | 4(2.2%) |  |
| Cholestatic liver disease,n(%) | 6(2.5%) | 3(1.6%) |  |
| Hepatolenticular degeneration, n(%) | 0 | 2(1.1%) |  |
| Budd-Chiari syndromen,(%) | 0 | 1(0.5%) |  |
| Unknown reason, n(%) | 35(14.8%) | 30(16.4%) |  |
| Classification of liver failure |  |  | 0.487 |
| ALF, n(%) | 30(12.7%) | 16(8.7%) |  |
| SALF, n(%) | 51(22.6%) | 42(23%) |  |
| CLF, n(%) | 27(11.4%) | 17(9.3%) |  |
| ACLF, n(%) | 128(54.2%) | 108(59%) |  |
| Hypertension, n(%) | 17(7.2%) | 15(8.2%) | 0.704 |
| diabetes mellitus, n(%) | 17(7.2%) | 20(10.9%) | 0.183 |
| Smoking, n(%) | 97(41.1%) | 76(41.5%) | 0.930 |
| Alcohol drinking, n(%) | 77(32.6%) | 58(31.7%) | 0.839 |
| Nausea and vomiting symptoms | 85(36.0%) | 45(24.6%%) | 0.012 |
| Complication |  |  |  |
| Bleeding, n(%) | 56(23.7%) | 60(32.8%%) | 0.040 |
| Ascites, n(%) | 143(60.6%) | 137(74.9%) | 0.002 |
| Secondary infection, n(%) | 171(72.5%) | 148(80.9%) | 0.045 |
| HE, n(%) | 42(17.8%) | 79(43.2%) | ＜0.001 |
| Hepatorenal syndrome, n(%) | 10(4.2%) | 26(14.2%) | ＜0.001 |
| Hepatopulmonary syndrome, n(%) | 0 | 2(1.1%) | 0.190 |
| Electrolyte disturbance, n(%) | 69(29.2%) | 91(49.7%) | ＜0.001 |
| Encephaledema, n(%) | 6(2.5%) | 11(6.0%) | 0.074 |
| Use of 5-HT3RAs | 82(34.7%) | 23(12.6%) | ＜0.001 |
| Times of 5-HT3RAs used |  |  | 0.003 |
| ＜2 times,n(%) | 206(87.3%) | 112(95.6%) |  |
| ≥2 times,n(%) | 30(12.7%) | 8(4.4%) |  |
| Artificial liver support treatment |  |  |  |
| PE, n(%) | 84(35.6%) | 73(39.9%) | 0.367 |
| PA, n(%) | 20(8.5%) | 20(10.9%) | 0.396 |
| CRRT, n(%) | 15(6.4%) | 29(15.8%) | 0.002 |
| Antiviral therapy, n(%) | 133(56.4%) | 95(51.9%) | 0.365 |
| Antiviral drugs |  |  | 0.447 |
| ETV, n(%) | 112(47.5%) | 80(43.7%) |  |
| TAF, n(%) | 3(1.3%) | 2(1.1%) |  |
| TDF, n(%) | 5(2.1%) | 1(0.5%) |  |
| Adefovir, n(%) | 0 | 2(1.1%) |  |
| Lamivudine, n(%) | 4(1.7%) | 4(2.2%) |  |
| Anti-infective treatment | 191(80.9%) | 164(89.6%) | 0.014 |
| MELD score | 19.06±6.18 | 25.79±8.29 | ＜0.001 |

Abbreviations: ACLF, acute on chronic liver failure; ALF, acute liver failure; CLF, chronic liver failure; CRRT, continuous renal replacement therapy; ETV, entecavir; HAV, hepatitis A virus; HBV, hepatitis B virus; HCV, hepatitis C virus; HE, hepatic encephalopathy; HEV, hepatitis E virus; 5-HT3RA, 5-HT3 receptor antagonist; MELD score, model for end-stage liver disease score; PA, plasma adsorption; PE, plasma exchange; SALF, subacute liver failure; SD, standard deviation; TAF, tenofovir alafenamide; TDF, tenofovir disoproxil fumarate.

Supplementary Table S6. Comparison of outcomes by unadjusted and adjusted analyses.

|  | Model | Use of 5-HT3RAs | HR(95%CI) | *p* value |
| --- | --- | --- | --- | --- |
| 28-day outcome | Unadjusted | No | Reference | ＜0.001 |
|  |  | Yes | 0.30(0.17,0.54) |  |
|  | Multivariate Cox regression | No | Reference | ＜0.001 |
|  |  | Yes | 0.18(0.10,0.34) |  |
|  | PSM | No | Reference | ＜0.001 |
|  |  | Yes | 0.10 (0.04,0.26) |  |
|  | IPTW | No | Reference | ＜0.001 |
|  |  | Yes | 0.18(0.08,0.39) |  |
| 90-day outcome | Unadjusted | No | Reference | ＜0.001 |
|  |  | Yes | 0.35(0.22,0.54) |  |
|  | Multivariate Cox regression | No | Reference | ＜0.001 |
|  |  | Yes | 0.21(0.13,0.33) |  |
|  | PSM | No | Reference | ＜0.001 |
|  |  | Yes | 0.16(0.08,0.31) |  |
|  | IPTW | No | Reference | ＜0.001 |
|  |  | Yes | 0.18(0.10,0.32) |  |

CI, confidence interval; HR, hazard ratio; 5-HT3RA, 5-HT3 receptor antagonist; IPTW, inverse probability treatment weighting; PSM, propensity score matching.
